# Analysis of the Rumen Microbiota of Beef Calves Supplemented During the Suckling Phase

**DOI:** 10.3389/fmicb.2019.01131

**Published:** 2019-05-28

**Authors:** Jeferson M. Lourenco, Todd R. Callaway, Troy J. Kieran, Travis C. Glenn, Joshua C. McCann, R. Lawton Stewart

**Affiliations:** ^1^Department of Animal and Dairy Science, University of Georgia, Athens, GA, United States; ^2^Department of Environmental Health Science, University of Georgia, Athens, GA, United States; ^3^Department of Animal Sciences, University of Illinois at Urbana–Champaign, Urbana, IL, United States

**Keywords:** 16S rRNA, creep feeding, exogenous feed enzymes, *Prevotella*, rumen microbiota, xylanase

## Abstract

A study was conducted to examine the effects of supplementing beef calves during their suckling phase (popularly known as creep feeding) with supplements that contained or did not contain the enzyme xylanase. Forty-two cow-calf pairs were divided into three groups and assigned to one of three treatments for a period of 105 days, as follows: (1) No supplemental feed for calves (control; CON); (2) Corn and soybean meal-based supplement feed for calves (positive control; PCON); and (3) Same feed regimen as PCON with xylanase added to the supplement (enzyme; ENZ). After 105 days, out of the 42 calves participating in the study, 25 male calves were randomly selected (8 from CON, 9 from PCON, and 8 from ENZ) and samples of their forestomach were collected by esophageal tubing. Immediately after this procedure, all calves were weaned, commingled, and placed in a common post-weaning diet for 4 weeks. At the end of this period, ruminal fluid was once again collected from the same 25 calves. All samples were subjected to DNA extraction and 16S rRNA gene sequencing. At weaning, most of the alpha diversity indexes were greater in CON; however, no differences (*P* ≥ 0.23) in alpha diversity were observed in samples collected 4 weeks after weaning. Regardless of treatment, 2 phyla – *Bacteroidetes* and *Firmicutes* – comprised approximately 80% of the total bacterial abundance of samples collected on both days. At the genus level, an effect of diet (*P* = 0.02) was observed for *Prevotella* in the samples collected at weaning; however, no differences were detected in the samples collected 4 weeks after weaning. Calf average daily gain (ADG) during the 105-day creep feeding trial tended (*P* = 0.09) to be greater in the groups that received supplementation, with the greatest numerical value observed in ENZ. Moreover, there was a positive correlation (ρ = 0.43; *P* = 0.03) between ADG and abundance of *Prevotella*, indicating the importance of this bacterial group for ruminants. In summary, most of the significant differences found in this study were detected at weaning, and the majority of them disappeared 4 weeks after the calves were weaned and commingled.

## Introduction

The practice of supplementing beef calves during their suckling stage is commonly known as creep feeding. Throughout the years, applied nutrition research has shown that this kind of supplementation can lead to improvement in calf growth rates (Prichard et al., 1989; Tarr et al., 1994; Viñoles et al., 2013). Previous studies have been conducted to improve the quality of creep feeds, and these include finding the optimum level of protein in such supplements (Lopes et al., 2014), and the addition of trace minerals to it (Moriel and Arthington, 2013); however, the inclusion of exogenous enzymes into creep feeds is something novel. Although exogenous feed enzymes are commonly used in swine and poultry rations, the use of these feed additives in ruminant diets is not very usual (Meale et al., 2014). Feed enzymes designed for ruminants usually contain xylanase and cellulase activities resulting from bacterial or fungal fermentations. These compounds can enhance fiber degradation in the rumen, resulting in improved feed efficiency (Beauchemin et al., 2003; He et al., 2014).

At birth, beef calves are not fully functional ruminants and the reticulo-rumen portion of their stomach represent less than 40% of the total stomach mass. However, as the reticulo-rumen is colonized by microbes and fermentation commences, it quickly becomes the dominant portion, and it comprises about 2/3 of the calf’s stomach at weaning (Church, 1979). This rapid development occurs because the mixed microbial fermentation begins as solid feed is introduced and this fermentation produces volatile fatty acids (VFA), which stimulate the growth of the ruminal epithelium (Anderson et al., 1987; Yáñez-Ruiz et al., 2015). Moreover, the composition of feeds consumed by calves directly influences their ruminal microbiota, ruminal epithelial development, and the density of papillae for VFA absorption as the calf nears weaning (Petri et al., 2013; Henderson et al., 2015).

Next generation sequencing techniques have revolutionized our understanding of the composition of the rumen microbiota (Neves et al., 2017). Combining these new techniques with more traditional microbiological methods and animal production parameters promises to produce significant advances in our knowledge of the function of the ruminal microbial ecosystem (Krause et al., 2013). While some associations between specific bacterial phylotypes and animal performance traits (such as feed efficiency) have been recognized (Hernandez-Sanabria et al., 2012; Myer et al., 2015; Shabat et al., 2016), no specific “best” microbial population has been identified. Furthermore, information is limited on the development of the ruminal microbial population of beef calves as they become fully functional ruminants, since most studies in this area have been conducted using dairy animals (Jami et al., 2013; Rey et al., 2014; Meale et al., 2016). Therefore, this study was carried out to investigate the effects of feeding beef calves different diets during the last portion of their suckling phase (i.e., the last 105 days prior to weaning), and to evaluate the effects of those diets on their ruminal microbiotas. Additionally, to verify if any differences would persist, calves were re-evaluated 4 weeks post-weaning.

## Materials and Methods

All procedures involving live animals were verified and approved by the University of Georgia’s Office of Animal Care and Use (Animal Use Protocol #A2015 07–018-Y1-A0). The cow-calf pairs used in this study were located at the University of Georgia Beef Research Unit in Eatonton, GA (33°24 N, 83°29 W).

### Animals and Diets Offered

Animal and diet management are described in Lourenço (2017). Briefly, a group of 42 suckling beef calves (36 males and 6 females; body weight = 177 ± 27 kg; age = 130 ± 19 days-old) and their respective dams were divided into three treatment groups. These groups were stratified by calf sex, body weight, and age, and were assigned to 1 of 3 treatments for a period of 105 days, as follows: (1) Control group with no supplementation of calves (CON); (2) Positive control group in which the calves were creep fed with a corn and soybean meal-based feed (PCON); and (3) Group with the same feed regimen as PCON but with the addition of the enzyme xylanase (ENZ). The feed used in the PCON and ENZ groups was made of 61.8% corn, 25.4% soybean meal, 7.0% salt, 3.6% limestone, 1.4% molasses, and 0.8% additional minerals and vitamins. The enzyme added to this feed in ENZ was included at 13,800 fungal xylanase units per kilogram of dry matter of the feed. This level of inclusion was used based on the results from a previous *in vitro* trial performed by our lab, which found this to be the level that optimizes dry matter digestibility (Lourenço, 2017). The commercial product (RONOZYME^®^ WX; DSM Nutritional Products) was granulated and had an average particle size of 600 μm. Its main activity was a heat-stable endo-1,4-β-xylanase from *Thermomyces lanuginosus* (EC number 3.2.1.8).

The experimental station in which the trial was conducted normally weans its calves when they reach 7 to 8 months of age. Given that long term creep feeding usually does not improve performance of calves due to their little interest in consuming solid feeds at younger ages (Prichard et al., 1989), and given that beef cows’ milk production is significantly reduced after approximately 100 days of lactation (National Research Council – NRC, 2000), we decided to supplement the calves after they reached about 130 days of age. Thus, the feeding trial took place when calves were on average 130 days old and lasted until they were weaned, 105 days later. The growth performance of all calves was monitored during the 105-day feeding trial by recording their body weights at the beginning and the end of the trial. Similarly, calf performance was monitored after weaning; however, calf growth rate was not the main trait evaluated in this study, since, in order to detect significant differences for this trait while having high statistical power, a substantially larger number of animals would have to be used in the study.

### Rumen Sampling Procedure

On the last day of the 105-day feeding trial, 25 male calves were selected (8 from CON, 9 from PCON, and 8 from ENZ) and their ruminal contents were sampled by esophageal tubing (described below). Immediately following collection, all calves were weaned, commingled, and placed on a common diet that consisted of bermudagrass pasture which was supplemented with 2.3 kg/day of a commercial feed (12% crude protein, 20% neutral detergent fiber, 2% fat). After being on this post-weaning common diet for 4 weeks, ruminal samples were once again collected from the same 25 male calves by esophageal tubing. Rumen fluid collection was performed similarly on both collection days. Briefly, ∼200 mL of ruminal contents was individually collected from each calf by esophageal tubing using a weighted metal perforated probe and an electric vacuum pump. Samples were placed into 15-mL sterile tubes and flash frozen by immersion in liquid nitrogen. They were then stored at –20°C. Frozen samples were later thawed, homogenized by inverting the tubes several times and vortexing for 5 s, and 0.5 mL of their liquid fraction was pipetted into plastic bead tubes. The samples were then further processed as described below to extract their DNA.

### DNA Extraction and Amplification

DNA was extracted from samples using the MoBio PowerSoil DNA isolation kit (MoBio Laboratories, Carlsbad, CA). This kit provides bacterial lysis by a combination of mechanical and chemical methods, and it was designed specifically for use with environmental samples and manure. In the first step, plastic bead tubes containing the samples were attached to a MoBio vortex adaptor and vortexed for 20 min. Next, 500 μL of samples were taken for the subsequent processes following manufacturer’s protocol (PowerSoil DNA isolation kit, Version 11212013). At the end of the protocol, 100 μl of molecular grade water was used to elute DNA from spin filter membranes, and 30 μL of DNA were transferred to a 96-well PCR plate.

PCR libraries were generated using the S-D-Bact-0341-b-S-17 (5′-CCTACGGGNGGCWGCAG-3′) forward and S-D-Bact-0785-a-A-21 (5′-GACTACHVGGGTATCTAATCC-3′) reverse primer pair (Klindworth et al., 2013) with some modifications (Wang et al., 2016; Kieran et al., 2017). Illumina TruSeq Read 1 to the forward and Illumina TruSeq Read 2 to the reverse primer were added on the 5′ ends. Additionally, 8 forward and 12 reverse fusion primers were synthesized, each with a unique variable length (5–8 bp) index sequence, between the 16S and TruSeq sequences (iTru-16S fusions).

DNA from each sample was amplified using two rounds of PCR. The first PCR used the iTru-16S fusions primers in 12.5-μl reactions using the KAPA HiFi Hotstart PCR kit (KAPA Biosystems, Wilmington, MA) using 2.5 μL of 5× Buffer, 0.375 μL of 10 mM dNTPs, 0.25 μL HotStart, 3.4 μL molecular grade water, 1 μL of 5 μM forward primer, 1 μL 5 μM reverse primer, and 4 μL of DNA. Thermocycler conditions were as follows: 95°C for 3 min, followed by 30 cycles of 95°C for 30 s, 55°C for 30 s, and 72°C for 30 s, with a final elongation step of 72°C for 5 min. PCR was performed using a T100 Thermal Cycler (BioRad, Hercules, CA) and amplicons were visualized using 1.5% gel electrophoresis. Three microliter of each PCR product were pooled and purified with SPRI-beads (Thermo Fisher Scientific, Waltham, MA) using a 0.92:1 ratio of beads to product pool. The second-round PCR primers consisted of Illumina TruSeqHT compatible 8 nucleotide indexed primers, iTru primers (Glenn et al., 2016). We used 25 μL reaction of KAPA HiFi HotStart Kits using 5 μL of 5× Buffer, 0.75 μL of 10 mM dNTPs, 0.5 μL HotStart, 3.75 μL molecular grade water, 2.5 μL of 5 μM forward primer, 2.5 μL 5 μM reverse primer, and 10 μL of purified iTru-16S amplicon pool. The following thermocycler conditions were used: 98°C for 2 min, followed by 10 cycles at 98°C for 30 s, 60°C for 30 s, 72°C for 30 s and a final extension at 72°C for 5 min. Library product was purified, and primers were removed with SPRI-beads (1:1 ratio) and pooled with other uniquely indexed samples prior to sequencing. Negative controls were used for both the extraction and PCR procedures.

### 16S rRNA Gene Sequencing and Analysis

All libraries were sent to the Georgia Genomics Facility^1^ for sequencing on an Illumina MiSeq using a v3 600 cycle kit (Illumina, San Diego, CA). Sequencing data were demultiplexed according to outer iTru indexes using bcl2fastq (Illumina, v1.8.4) to identify the sample pool. The iTru-16S amplicon pool was demultiplexed by internal barcodes to identify individual samples and primers were removed using Mr. Demuxy v1.2.0^2^. Paired-end sequencing reads were imported into Geneious v10.0.9 (Biomatters Limited, NJ), set as paired-reads with an expected insert size of 400 bp, and trimmed to remove low quality bases using default settings and a quality score of 0.001. Paired-end sequencing reads were then merged using the FLASH v1.2.9 plugin (Magoc and Salzberg, 2011). Data were exported from Geneious as FASTA files for further analysis using software package QIIME v1.9.1 (Caporaso et al., 2010). Operational taxonomic units (OTU) were clustered at 97% similarity with UCLUST, and cluster sequences were aligned to the Greengenes database (gg_13_8_otus). Singleton OTUs and OTUs whose representative sequences could not be aligned with PyNAST were removed. Alpha and beta diversities, and OTU richness were calculated after sample sizes were standardized to 6,040 sequences for samples collected on weaning day, and to 3,484 sequences for samples collected 4 weeks after weaning. These values were chosen because they allowed elimination of sampling depth heterogeneity without missing any sample collected on both days. The sequencing data for both collection days can be found on the MG-RAST website^3^ under accession numbers 4836929.3 and 4836938.3.

Alpha diversity indexes were computed using QIIME’s “alpha_rarefaction.py” script to determine Shannon diversity index, Simpson’s diversity index, Chao1, Faith’s Phylogenetic Diversity, and number of observed OTUs. Beta diversity between all pairs of samples was calculated using QIIME’s “beta_diversity_through_plots.py” script and results were visualized using 2-dimensional plots (Supplementary Figures S3, S4). Weighted UniFrac distances were used for the beta diversity plots. This metric was chosen because it accounts for phylogenetic relationships when measuring beta diversity (Lozupone and Knight, 2005; Hamady and Knight, 2009).

### Statistical Analysis

Statistical analyses were performed using QIIME scripts (QIIME pipeline v1.9.1; Caporaso et al., 2010), the software R (R Foundation for Statistical Computing, Vienna, Austria), and Minitab^®^ (v18.1). As can be seen in Supplementary Figures S6–S10, the taxa relative abundances, alpha-diversity indexes, and animal performance data were normally distributed. Except for the pre- versus post-weaning comparisons, the results were individually analyzed within each collection day (i.e., at weaning of 4 weeks post-weaning).

**FIGURE 1:**
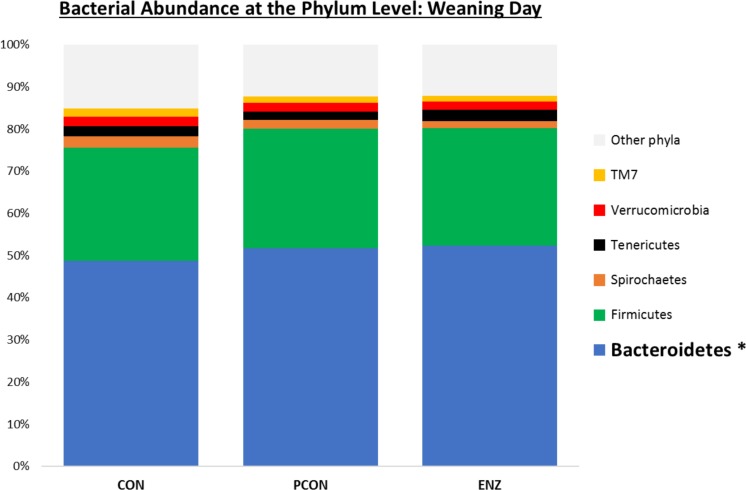
Relative bacterial abundance at the phylum level for calves in different diets (CON = conventional cow-calf system without supplementation of calves. PCON = calves were creep fed. ENZ = calves were creep fed with an enhanced feed containing xylanase): phyla with relative abundances ≥ 1.4% in the samples collected on calves’ weaning day (group averages shown). ^*^Bonferroni-corrected pairwise comparisons revealed differences across groups only for *Bacteroidetes*: Mean relative abundances (and standard deviations) were 48.8(2.4)^b^, 51.7(1.7)^a^ and 52.3(2.1)^a^ % for CON, PCON and ENZ, respectively, and means not sharing the same superscript ^a,b^ were different (*P* = 0.01).

**FIGURE 2:**
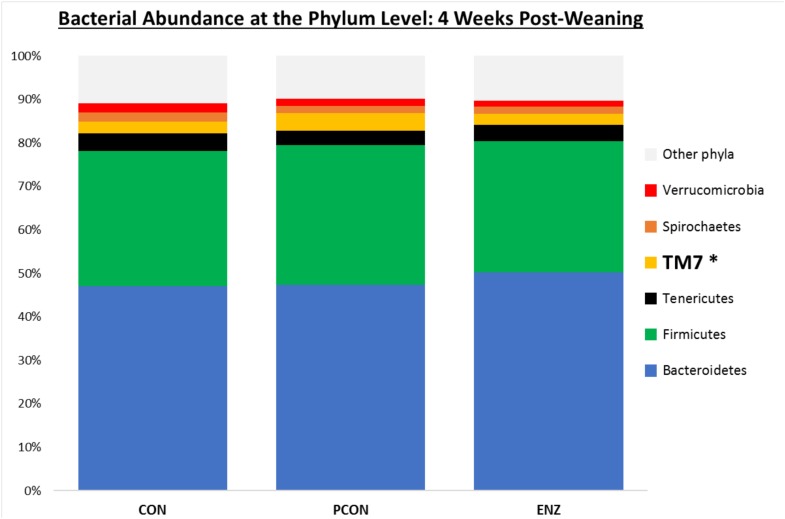
Relative bacterial abundance at the phylum level for calves in different diets (CON = conventional cow-calf system without supplementation of calves. PCON = calves were creep fed. ENZ = calves were creep fed with an enhanced feed containing xylanase): phyla with relative abundances ≥ 1.4% in the samples collected 4 weeks after weaning (group averages shown). ^*^Bonferroni-corrected pairwise comparisons revealed differences across groups only for TM7: Mean relative abundances (and standard deviations) were 2.8(0.74)^ab^, 4.1(1.5)^a^ and 2.5(0.57)^b^ % for CON, PCON and ENZ, respectively, and means not sharing the same superscript ^a,b^ were different (*P* = 0.01).

**FIGURE 3:**
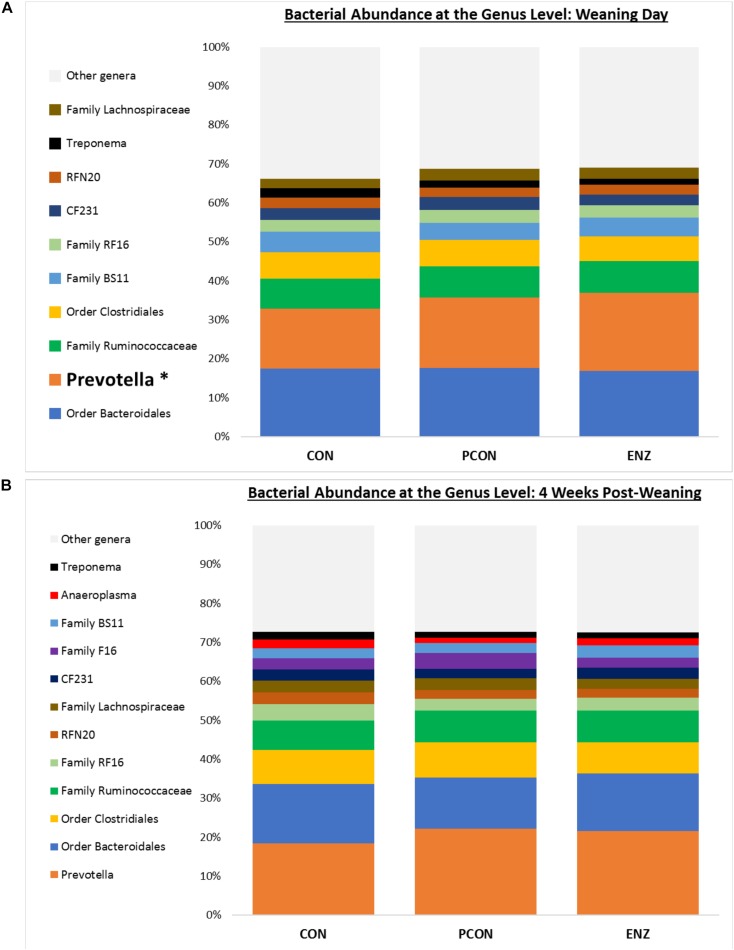
Bacterial abundance at the genus level for calves in different diets (CON = conventional cow-calf system without supplementation of calves. PCON = calves were creep fed. ENZ = calves were creep fed with an enhanced feed containing xylanase): genera with relative abundances ≥ 2% (group averages shown). **(A)** Samples collected on calves’ weaning day. **(B)** Samples collected 4 weeks after weaning. ^*^Bonferroni-corrected pairwise comparisons revealed differences across groups only for *Prevotella* on weaning day: Mean relative abundances (and standard deviations) were 15.4(1.4)^b^, 18.1(3.9)^ab^ and 20.1(3.4)^a^ % for CON, PCON and ENZ, respectively. Four weeks post-weaning, only a trend (*P* = 0.06) for differences in *Prevotella* were observed: Mean relative abundances were 18.3(2.7)^a^, 22.1(3.7)^a^ and 21.6(3.2)^a^ % for CON, PCON and ENZ, respectively. Means not sharing the same superscript ^a,b^ were different (*P* = 0.02).

Analysis of the alpha-diversity metrics and the growth performance of calves across groups were carried out by ANOVA using diet as a factor, and initial age was used as a covariate for the calf growth performance traits. Comparisons between all pairs of groups were performed using Fisher’s Least Significant Difference method. All the pairwise comparisons involving taxa (e.g., Tables 3, 4 and Figures 1–3) had their significance levels corrected by the Bonferroni’s method for multiple comparisons. This procedure was used for controlling the higher error rates normally associated with multiple comparisons, since the probability of a Type I error (false positive) increases as the number of hypotheses checked simultaneously increases. In the same way, the differences in beta diversity (Supplementary Figures S3, S4) were accessed using two-sample *t*-tests in which the *P*-values were corrected by Bonferroni’s method. Regardless of the statistical tests used, results were considered significant at *P* ≤ 0.05.

## Results and Discussion

The interested reader can refer to Supplementary Tables S1–S7 for additional information about the nutritive values of the pasture and the supplement offered to the calves, as well as the bacterial abundances at the taxonomic levels not presented here. After quality control and singleton removal, the samples obtained on weaning day yielded a total of 306,414 cleaned reads, resulting in an average of 12,257 reads per sample, with the number of sequences ranging from 6,040 to 17,627 (median = 13,509). For the samples obtained 4 weeks after calves were weaned, a total of 224,445 cleaned reads were observed, resulting in an average of 8,978 reads per sample, and the number of sequences ranging from 3,484 to 13,683 (median = 9,341). In the present study, we chose the lowest values observed in each collection day (i.e., 6,040 and 3,484 sequences for samples from weaning day and 4 weeks post-weaning, respectively) because they allowed elimination of sampling depth heterogeneity while keeping all collected samples in the calculations. However, this attempt to keep all samples in the calculations resulted in a relatively low yield of sequences observed. Still, for the purpose of this study, the Shannon and Simpson rarefaction plots (Supplementary Figures S1, S2) demonstrate that the sequencing depths chosen for both collection days were adequate, considering that these rarefaction plots clearly reached a plateau at the chosen depths. Haegeman et al. (2013) have established Shannon and Simpson as the best indexes to quantify and compare microbial taxonomic diversity, which gives support for the findings presented here. However, the rarefaction plots for the other metrics (e.g., observed OTUs and Chao1) indicates that some OTUs were likely eliminated due to our pre-established cutoff values. Since these metrics are skewed toward low-abundance OTUs, we may have missed several of them, and comparisons of low-abundant OTUs was compromised, which is a limitation of the present study.

### Alpha and Beta Diversities

Table 1 summarizes the microbial richness and diversity metrics calculated for samples collected on weaning day. Except for the Simpson index (*P* = 0.12), all of the richness and diversity metrics were greater (*P* ≤ 0.05) in CON compared to the other two treatments. The number of observed OTUs was also higher (*P* = 0.02) in the rumen samples obtained from calves in CON. Moreover, when comparing the two treatments in which calves were supplemented (i.e., PCON versus ENZ) no differences (*P* ≥ 0.95) were detected for any of the alpha diversity metrics. Even though each alpha diversity index shown here has its own strengths and limitations, no conclusions should be drawn based on only one single index. Instead, a better description of communities is achieved when multiple indexes are considered simultaneously (Morris et al., 2014). Thus, as can be noticed in the present study, although the differences in the diversity indexes between groups were not of enormous magnitude, most of those differences were statistically significant. This fact can be attributed to the very low diversity observed within each group of calves, causing the variation that occurred between groups to be significant. This peculiarity is only possible for animals living in similar environmental conditions, which was the case of the present study: not only the different cow-calf groups had their own paddocks, but each paddock had its own feeder and waterer, and the groups remained physically separated by double fences for the entire 105-day feeding trial (Supplementary Figure S5). The overall greater richness and diversity observed in the microbiome of CON calves indicates that supplementing calves for a period of 105 days may contribute to a reduction in microbial richness and diversity due to supplementation, generating some important differences between the microbiota of supplemented and non-supplemented calves. Additionally, the lack of significant differences in diversity between PCON and ENZ indicates that the presence of xylanase in the supplement did not affect the overall ruminal microbial richness and diversity during the 105-day feeding trial.

**TABLE 1:** Effect of treatment on richness and alpha diversity at 97% similarity after rarefaction to 6,040 sequences per sample for samples collected on weaning day.

	**Treatment^1^**				
**Item**	**CON**	**PCON**	**ENZ**	**SE**	***P*-value**
Shannon index	10.4^a^	10.2^b^	10.2^b^	0.09	0.04
Simpson index	0.9981	0.9976	0.9976	0.00	0.12
Chao1	7,082^a^	6,729^ab^	6,626^b^	181.00	0.05
PD whole tree^2^	159.0^a^	151.8^b^	150.1^b^	2.63	0.01
Observed OTUs	2,572^a^	2,421^b^	2,424^b^	55.50	0.02

*^1^CON = conventional cow-calf system without supplementation of calves. PCON = calves were creep fed. ENZ = calves were creep fed with an enhanced feed containing xylanase; ^2^Faith’s Phylogenetic Diversity. ^a,b^Means within a row with different superscripts differ (*P* ≤ 0.05) according to Fisher’s Least Significance Difference method.*

Table 2 shows alpha diversity metrics for the samples collected 4 weeks after calves were weaned. In contrast to the initial sampling time, samples collected after weaning revealed no differences (*P* ≥ 0.23) for any of the metrics calculated. Thus, although there were differences at the time of weaning, such differences were not maintained after the calves were weaned and placed in the same feed regimen for 4 weeks. Regarding beta diversity, results computed using the weighted UniFrac distance matrices did not show any differences due to diet both on weaning day (*P* ≥ 0.99) and 4 weeks after weaning (*P* ≥ 0.11). Principal coordinate analysis of beta diversity for both sampling days are shown in Supplementary Figures S3, S4.

**TABLE 2:** Effect of treatment on richness and alpha diversity at 97% similarity after rarefaction to 3,484 sequences per sample for samples collected 4 weeks after weaning.

	**Treatment^1^**				
**Item**	**CON**	**PCON**	**ENZ**	**SE**	***P*-value**
Shannon index	9.8	9.8	9.7	0.09	0.58
Simpson index	0.9973	0.9974	0.9972	0.00	0.81
Chao1	4,316	4,239	4,108	158.00	0.45
PD whole tree^2^	104.3	103.5	100.7	2.09	0.23
Observed OTUs	1,592	1,609	1,555	45.30	0.50

*^1^CON = conventional cow-calf system without supplementation of calves. PCON = calves were creep fed. ENZ = calves were creep fed with an enhanced feed containing xylanase; ^2^Faith’s Phylogenetic Diversity. None of the means within each row were significantly different (*P* ≥ 0.05) according to Fisher’s Least Significant Difference method.*

### Bacterial Abundance

Bacterial relative abundance tables for both collection days at different taxonomic levels are shown in Figures 1–3. Regardless of diet or collection day, the phylum detected at the greatest abundance in the rumen fluid of calves was *Bacteroidetes* (47.1–52.3% relative abundance). The second most abundant phylum was *Firmicutes*, with abundances varying between 26.8 and 32.2%. These findings are similar to those reported by McCann et al. (2014) and Myer et al. (2015), who also found a predominance of *Bacteroidetes* (53–78%), followed by *Firmicutes* (15–33%) in the rumen fluid of cattle. However, when analyzing the solid instead of the liquid fraction of ruminal digesta, McCann et al. (2016) reported that *Firmicutes* was more predominant than *Bacteroidetes*. Nevertheless, the authors also analyzed the liquid fraction of their samples, and in such samples, they found results more similar to ours for the 2 predominant phyla: their relative abundance of *Bacteroidetes* ranged from 59 to 65%, and of *Firmicutes* from 28–31%. Hence, the portion of the rumen digesta (liquid or solid) used in microbiome analysis has a significant impact on the bacterial abundances reported.

**FIGURE 4:**
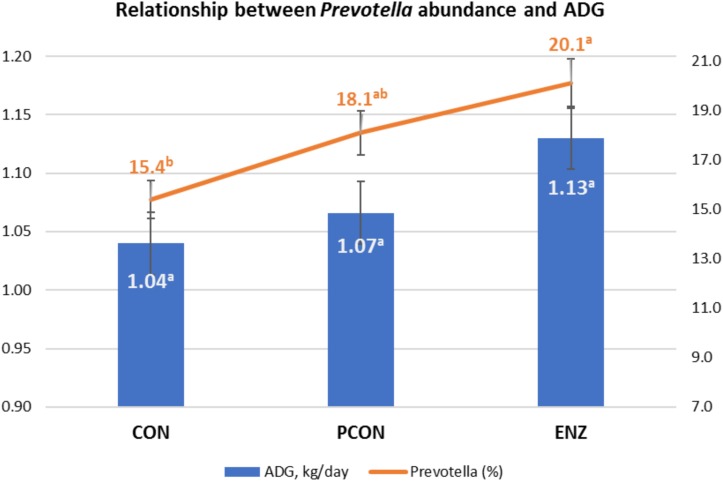
Graphical relationship between relative abundance of the genus *Prevotella* and average daily gain (ADG) during the 105-day creep feeding trial. An effect of diet was observed for abundance of *Prevotella* (*P* = 0.02), and a trend (*P* = 0.09) for ADG. Spearman correlation coefficient showed a positive correlation between ADG and *Prevotella* (ρ = 0.43; *P* = 0.03). CON = conventional cow-calf system without supplementation of calves. PCON = calves were creep fed. ENZ = calves were creep fed with an enhanced feed containing xylanase.

As seen in Figure 1, on weaning day, an effect of diet on bacterial abundance at the phylum level was observed only for *Bacteroidetes*, whose relative abundance was greatest (*P* = 0.01) in the 2 groups of calves that received feed supplementation: PCON and ENZ. When comparing the samples collected 4 weeks after weaning (Figure 2), differences were detected only for the phylum TM7, which had the greatest (*P* = 0.01) abundance observed in PCON. At the genus level (Figure 3), in the samples collected at weaning, an effect of diet was observed for *Prevotella*, whose relative abundance was lowest (*P* = 0.02) in CON. However, in the samples collected 4 weeks after weaning, no differences due to diet were detected (*P* ≥ 0.06; Figures 3, 4). Several studies have assessed the abundance of *Prevotella* in ruminants. Carberry et al. (2012) found a lower abundance of *Prevotella* in the rumen fluid of beef cattle receiving a high-forage diet. Similarly, Petri et al. (2013) reported a linear increase in *Prevotella* abundance as Angus heifers transitioned from a diet composed of 95% grass hay to a finishing diet containing 9% forage. Analogous results have also been observed in sheep: Bekele et al. (2010) fed 2 distinct diets – one containing 33% and another with 91% forage – to fistulated sheep and found that animals receiving the diet with the higher percentage of forage had a lower abundance of *Prevotella* in their rumen fluid. These mentioned findings are in alignment with our results, given that all animals in CON received only forage as their source of solid feed and ended up having the lowest relative abundance of *Prevotella* among all diets.

Tables 3, 4 show the effect of time (or weaning) on each of the diets tested in our study. At the phylum level, regardless of diet, the abundance of *Bacteroidetes*, *Spirochaetes*, and *Verrucomicrobia* numerically decreased 4 weeks after the calves were weaned. However, this decrease was significant (*P* = 0.03) only for the phyla *Bacteroidetes* in the PCON group (Table 3). Conversely, the abundance of *Firmicutes*, *Tenericutes*, and TM7 numerically increased in all treatment groups, but these shifts were statistically significant (*P* ≤ 0.03) only for *Tenericutes* from calves in CON, and for TM7 from calves in the PCON group. Similarly, at the genus level, the abundance of the genera *Prevotella*, *Ruminococcus*, *Anaeroplasma*, *Succiniclasticum*, *Butyrivibrio*, and *Coprococcus* numerically increased as the calves aged, but just a few of those shifts were statistically significant, and they did not follow a clear pattern regarding calf supplementation (Table 4). Jami et al. (2013) suggested that the rumen bacterial community is not exclusively influenced by diet, but also by the age of the animals. For instance, they found that abundance of *Tenericutes* and TM7 are greater in older animals, which is somewhat in line with our findings for these two phyla.

**TABLE 3:** Effect of treatment on relative abundance of the main phyla^*^ on weaning day versus 4 weeks after (group averages shown).

	**Treatment^1^**									
	**CON**			**PCON**			**ENZ**		
**Phyla**	**At weaning^2^**	**Post-weaning^3^**	**Contrast^4^**	**At weaning^2^**	**Post-weaning^3^**	**Contrast^4^**	**At weaning^2^**	**Post-weaning^3^**	**Contrast^4^**
*Bacteroidetes*	48.8	47.1	0.99	51.7	47.3	0.03	52.3	50.3	0.99
*Firmicutes*	26.8	31.0	0.13	28.4	32.2	0.19	27.8	30.2	0.99
*Spirochaetes*	2.7	2.1	0.99	2.0	1.5	0.99	1.8	1.7	0.99
*Tenericutes*	2.4	4.0	0.03	2.0	3.3	0.07	2.6	3.8	0.29
*Verrucomicrobia*	2.2	2.1	0.99	2.1	1.6	0.99	2.0	1.4	0.99
TM7	1.8	2.8	0.26	1.5	4.1	0.01	1.4	2.5	0.14

^*^Phyla with relative abundance across treatments ≥1.4%. ^1^CON = conventional cow-calf system without supplementation of calves. PCON = calves were creep fed. ENZ = calves were creep fed with an enhanced feed containing xylanase; ^2^At weaning: ruminal samples collected on weaning day; ^3^Post-weaning: ruminal samples collected 4 weeks after calves were weaned; ^4^Contrast: *P*-value calculated using the Bonferroni-corrected pairwise comparison within treatment for “At weaning” vs. “Post-weaning.”

**TABLE 4:** Effect of treatment on relative abundance of the main genera^*^ on weaning day versus 4 weeks after (group averages shown).

	**Treatment^1^**									
	**CON**			**PCON**			**ENZ**		
**Genera**	**At weaning^2^**	**Post-weaning^3^**	**Contrast^4^**	**At weaning^2^**	**Post-weaning^3^**	**Contrast^4^**	**At weaning^2^**	**Post-weaning^3^**	**Contrast^4^**
*Prevotella*	15.4	18.3	0.99	18.1	22.1	0.16	20.1	21.6	0.99
CF231	3.0	2.9	0.99	3.3	2.4	0.20	2.7	2.9	0.99
RFN20	2.7	3.1	0.99	2.3	2.2	0.99	2.5	2.3	0.99
*Treponema*	2.4	2.0	0.99	1.8	1.5	0.99	1.6	1.6	0.99
YRC22	1.3	1.1	0.99	1.6	1.3	0.99	1.2	1.8	0.24
*Pseudobutyrivibrio*	1.1	1.1	0.99	1.2	1.1	0.99	1.1	1.5	0.99
*Ruminococcus*	1.1	1.6	0.11	1.1	1.7	0.03	0.9	1.3	0.72
*Anaeroplasma*	0.8	2.2	0.01	0.8	1.5	0.76	1.0	1.8	0.59
*Succiniclasticum*	0.6	1.1	0.55	0.9	1.3	0.73	0.9	1.2	0.99
*Methanobrevibacter*	0.5	0.7	0.99	0.6	0.7	0.99	1.3	0.8	0.99
*Butyrivibrio*	0.5	0.8	0.10	0.7	0.8	0.99	0.6	0.7	0.99
*Coprococcus*	0.4	0.7	0.01	0.5	0.8	0.01	0.5	0.7	0.56

^*^Genera that were individually identified and were present at ≥0.5% in at least one treatment group. ^1^ CON = conventional cow-calf system without supplementation of calves. PCON = calves were creep fed. ENZ = calves were creep fed with an enhanced feed containing xylanase; ^2^ At weaning: ruminal samples collected on weaning day; ^3^ Post-weaning: ruminal samples collected 4 weeks after calves were weaned; ^4^ Contrast: *P*-value calculated using the Bonferroni-corrected pairwise comparison within treatment for “At weaning” vs. “Post-weaning.”

### Calf Growth Performance-Microbiota Relationship

As previously stated, evaluating the growth performance of calves was not the main objective of the present study. Still, those results were collected, statistically analyzed, and are presented in Table 5. It can be noticed from that table that numerical differences (*P* = 0.09) were observed for calves in the different treatment groups during the 105-day feeding trial. On the other hand, average daily gains during the first 4 weeks after calves were weaned and placed in a common diet were more similar across treatments (*P* = 0.59). Consequently, the plane of nutrition to which the calves were exposed during their suckling phase had no significant impact on their post-weaning performance. However, the biological reasons for the greater significance in average daily gains observed during the suckling phase might be due to a combination of factors, including the differences observed in the calves’ rumen microbiota. While microbial richness and diversity, assessed at the end of the suckling period, tended to be lower in the supplemented groups (PCON and ENZ), such differences were equalized 4 weeks after weaning. Previous research has demonstrated that a lower richness of microbiome gene content and taxa is correlated to better feed efficiency. Shabat et al. (2016) demonstrated that, although the most efficient rumen microbiomes had lower richness and diversity, they produced larger amounts of relevant output metabolites to meet the animal’s energetic needs. Thus, efficient microbiomes appear to be less complex, but more specialized at supporting the animal’s energy requirements. Although the differences in average daily gain during our feeding trial were not statistically significant (*P* = 0.09), the way in which their numerical variance occurred are in line with the findings from Shabat et al. (2016). Another factor that was likely associated with the numerical differences in calf average daily gain was the abundance of *Prevotella*. Calves in the CON group had the lowest numerical average daily gain and the lowest (*P* = 0.02) abundance of this genus of bacteria. Moreover, Spearman’s correlation coefficient calculated for the abundance of *Prevotella* and average daily gain during the feeding trial showed a positive (ρ = 0.43; *P* = 0.03) correlation between these two factors. *Prevotella* has been regarded as being important in degradation of fiber (Bekele et al., 2010), as well as in the metabolization of starch, peptides, and pectin (Carberry et al., 2012). Therefore, the abundance of *Prevotella* may have also contributed to the numerical differences observed in calf growth.

**TABLE 5:** Initial age, weight, and average daily gain (ADG) of calves during the 105-day creep feeding trial (last third of their suckling phase) and during the first 4 weeks after weaning^*^.

	**Treatment^1^**				
**Item**	**CON**	**PCON**	**ENZ**	**SE**	***P*-value**
Initial age, days	135 (20)	127 (20)	128 (21)	9.80	0.68
Initial weight, kg	194.8 (29)	181.4 (24)	177.7 (30)	13.2	0.42
ADG Period1, kg/day^2^	1.04 (0.11)	1.07 (0.12)	1.13 (0.12)	0.04	0.09
ADG Period2, kg/day^3^	0.85 (0.22)	0.93 (0.34)	1.02 (0.53)	0.16	0.59

^1^CON = conventional cow-calf system without supplementation of calves. PCON = calves were creep fed. ENZ = calves were creep fed with an enhanced feed containing xylanase; ^2^ADG Period1 = ADG during the 105-day feeding trial (last third of suckling phase); ^3^ADG Period2 = ADG during the first 4 weeks after weaning. ^*^Results shown as mean and standard deviations (in parenthesis). None of the means within each row were significantly different (*P* ≥ 0.05) according to Fisher’s Least Significant Difference test.

In summary, the present study had some limitations regarding sample size since only 25 calves participated in the microbiome evaluations. In addition, comparisons of low-abundant OTUs were compromised due to the chosen sampling depths. Despite these limitations, some differences in the calves’ ruminal microbiota were observed due to the different supplementation strategies used during their suckling phase. However, most of those differences (especially the ones regarding diversity) were mitigated after the calves were weaned and fed a common diet for a period of 4 weeks, indicating that commingling and feeding calves a common diet for 4 weeks is apparently enough to equalize the diversity in their ruminal microbial populations. Another important finding from the present study was the positive correlation (*P* = 0.03) between the abundance of *Prevotella* and calf average daily gain during the suckling phase, indicating that bacteria from this genus likely play an important role in the ruminal microbiota of young beef calves, and contribute to their growth in a positive manner. Still, further research is necessary to elucidate *Prevotella*’s specific mode of action in the rumen microbiota of suckling beef calves.

## Ethics Statement

All procedures involving live animals were verified and approved by the University of Georgia’s Office of Animal Care and Use (Animal Use Protocol #A2015 07–018-Y1-A0).

## Author Contributions

All authors listed have made a substantial, direct and intellectual contribution to the work, and approved it for publication.

## Conflict of Interest Statement

The authors declare that the research was conducted in the absence of any commercial or financial relationships that could be construed as a potential conflict of interest.

## Footnotes

1 http://dna.uga.edu

2 https://pypi.python.org/pypi/Mr_Demuxy/1.2.0

3 https://www.mg-rast.org
