# Supplementary material for: Analysis of the Rumen Microbiota of Beef Calves Supplemented During the Suckling Phase
Source: Front Microbiol. 2019 May 28;10:1131. doi: 10.3389/fmicb.2019.01131 (PMC6547912; doi:10.3389/fmicb.2019.01131)
Supplement: Supplementary file 1 [file Presentation_1.pdf]

SUPPLEMENTAL MATERIAL

Table S1. Analyzed nutrient content of the grass and plain supplement offered to calves.

**Analyzed Nutrient Content (dry matter basis)**	**GRASS**	**SUPPLEMENT**
Total Digestible Nutrients, %	56.0	69.0
Crude Protein, %	12.0	18.9
Neutral Detergent Fiber, %	73.39	7.66
Acid Detergent Fiber, %	36.40	3.70
Calcium, %	0.50	2.32
Phosphorus, %	0.36	0.51
Magnesium, %	0.16	0.17
Digestible Energy, Mcal/kg	2.47	3.04

Table S2. Effect of treatment on relative abundance of bacteria classes for samples collected on weaning day (averages shown).

	Treatment[1]			Bonferroni
**Classes**	**CON**	**PCON**	**ENZ**	***P*-value[2]**
Bacteroidia	48.8	51.7	52.3	0.17
Clostridia	23.9	25.7	25.0	0.58
Erysipelotrichi	2.9	2.5	2.7	0.88
Spirochaetes	2.6	2.0	1.7	0.34
Verruco-5	2.1	2.0	1.9	0.99
Mollicutes	2.0	1.7	2.4	0.27
TM7-3	1.8	1.5	1.4	0.25
Lentisphaeria	1.8	1.2	0.7	0.17
4C0d-2	1.7	0.9	0.5	0.20
Alphaproteobacteria	0.8	0.6	0.7	0.40
Thermoplasmata	0.7	0.4	0.5	0.21
Methanobacteria	0.6	0.7	1.4	0.47
Gammaproteobacteria	0.5	0.6	0.3	0.25
Other classes	2.7	2.6	2.2	≥ 0.17
Unassigned OTUs	7.2	5.9	6.2	0.21

[1] CON = conventional cow-calf system without supplementation of calves. PCON = calves were creep fed. ENZ = calves were creep fed with an enhanced feed containing xylanase.

[2] *P*-values based on Bonferroni's method for multiple comparisons.

Table S3. Effect of treatment on relative abundance of bacteria orders for samples collected on weaning day (averages shown).

	Treatment[1]			Bonferroni
**Orders**	**CON**	**PCON**	**ENZ**	***P*-value[2]**
Bacteroidales	48.8	51.7	52.3	0.21
Clostridiales	23.9	25.7	25.0	0.67
Erysipelotrichales	2.9	2.5	2.7	0.92
Spirochaetales	2.5	1.9	1.6	0.39
WCHB1-41	2.0	1.9	1.8	0.98
CW040	1.8	1.5	1.4	0.30
YS2	1.7	0.9	0.5	0.25
Victivallales	1.6	1.1	0.7	0.21
Anaeroplasmatales	1.0	0.9	1.2	0.86
RF39	0.9	0.6	0.9	0.30
E2	0.7	0.4	0.5	0.28
Methanobacteriales	0.6	0.7	1.4	0.61
Rickettsiales	0.6	0.4	0.6	0.67
Aeromonadales	0.5	0.5	0.2	0.46
Other orders	3.5	3.2	3.0	≥ 0.21
Unassigned OTUs	7.2	5.9	6.2	0.28

[1] CON = conventional cow-calf system without supplementation of calves. PCON = calves were creep fed. ENZ = calves were creep fed with an enhanced feed containing xylanase.

[2] *P*-values based on Bonferroni's method for multiple comparisons.

Table S4. Effect of treatment on relative abundance of bacteria families for samples collected on weaning day (averages shown).

Families	Treatment[1] CON	PCON	ENZ	Bonferroni *P*-value[2]
Unidentified family 1[A]	17.4	17.6	16.8	0.98
Prevotellaceae	15.5	18.3	20.2	0.20
Ruminococcaceae	8.9	9.2	9.3	0.64
Unidentified family 2[B]	6.8	6.8	6.2	0.60
Paraprevotellaceae	6.0	6.5	5.1	0.17
BS11	5.3	4.3	4.8	0.70
Lachnospiraceae	4.8	5.9	5.6	0.40
RF16	3.1	3.3	3.2	0.98
Erysipelotrichaceae	2.9	2.5	2.7	0.93
Spirochaetaceae	2.5	1.9	1.6	0.41
RFP12	1.9	1.7	1.7	0.97
F16	1.8	1.5	1.4	0.31
Unidentified family 3[C]	1.7	0.9	0.5	0.25
Victivallaceae	1.6	1.1	0.7	0.20
Veillonellaceae	1.6	1.9	1.9	0.63
Anaeroplasmataceae	1.0	0.9	1.2	0.88
Unidentified family 4[D]	0.9	0.6	0.9	0.31
Mogibacteriaceae	0.8	0.9	1.0	0.63
Bacteroidaceae	0.8	0.8	0.5	0.20
Methanomassiliicoccaceae	0.7	0.4	0.5	0.30
Methanobacteriaceae	0.6	0.7	1.4	0.60
Unidentified family 5[E]	0.6	0.4	0.6	0.64
Clostridiaceae	0.5	0.7	0.5	0.25
S24-7	0.5	0.7	1.2	0.25
Succinivibrionaceae	0.5	0.5	0.2	0.46
Other families	4.2	3.9	4.0	≥ 0.20
Unassigned OTUs	7.2	5.9	6.2	0.29

[1] CON = conventional cow-calf system without supplementation of calves. PCON = calves were creep fed. ENZ = calves were creep fed with an enhanced feed containing xylanase.

[2] *P*-values based on Bonferroni's method for multiple comparisons.

[A]Order *Bacteroidales*; [B]Order *Clostridiales*; [C]Order YS2; [D]Order RF39; [E]Order *Rickettsiales*.

Table S5. Effect of treatment on relative abundance of bacteria classes for samples collected 4 weeks after weaning (averages shown).

Classes	Treatment[1] CON	PCON	ENZ	Bonferroni *P*-value[2]
Bacteroidia	47.1	47.3	50.2	0.60
Clostridia	27.6	29.6	27.5	0.83
Mollicutes	3.6	3.1	3.4	0.96
Erysipelotrichi	3.3	2.4	2.5	0.96
TM7-3	2.8	4.1	2.5	0.33
Spirochaetes	2.1	1.5	1.6	0.60
Verruco-5	2.0	1.6	1.3	0.83
Unidentified class 1[A]	0.9	1.1	1.3	0.96
Methanobacteria	0.8	0.8	0.9	0.96
Gammaproteobacteria	0.6	0.5	0.5	0.96
4C0d-2	0.6	0.6	0.6	0.98
Alphaproteobacteria	0.6	0.6	0.6	0.98
Fibrobacteria	0.6	0.5	0.4	0.60
Lentisphaeria	0.6	0.5	0.7	0.96
Deltaproteobacteria	0.5	0.4	0.4	0.98
Other classes	1.8	1.6	1.9	≥ 0.24
Unassigned OTUs	4.4	3.8	3.6	0.24

[1] CON = conventional cow-calf system without supplementation of calves. PCON = calves were creep fed. ENZ = calves were creep fed with an enhanced feed containing xylanase.

[2] *P*-values based on Bonferroni's method for multiple comparisons.

[A] Phylum SR1.

Table S6. Effect of treatment on relative abundance of bacteria orders for samples collected 4 weeks after weaning (averages shown).

Orders	Treatment[1] CON	PCON	ENZ	Bonferroni *P*-value[2]
Bacteroidales	47.1	47.3	50.2	0.69
Clostridiales	27.6	29.6	27.5	0.78
Erysipelotrichales	3.3	2.4	2.5	0.87
CW040	2.8	4.1	2.5	0.41
Anaeroplasmatales	2.5	1.7	2.0	0.78
Spirochaetales	2.0	1.5	1.6	0.70
WCHB1-41	1.9	1.5	1.2	0.83
Unidentified order 1[A]	0.9	1.1	1.3	0.95
RF39	0.9	1.2	1.3	0.86
Methanobacteriales	0.8	0.8	0.9	0.95
YS2	0.6	0.6	0.6	0.99
Fibrobacterales	0.6	0.5	0.4	0.70
Victivallales	0.5	0.5	0.7	0.95
Other orders	4.0	3.5	3.8	≥ 0.27
Unassigned OTUs	4.4	3.8	3.6	0.27

[1] CON = conventional cow-calf system without supplementation of calves. PCON = calves were creep fed. ENZ = calves were creep fed with an enhanced feed containing xylanase.

[2] Test *P*-values based on Bonferroni's method for multiple comparisons.

[A] Phylum SR1.

Table S7. Effect of treatment on relative abundance of bacteria families for samples collected 4 weeks after weaning (averages shown).

	Treatment[1]			Bonferroni
**Families**	**CON**	**PCON**	**ENZ**	***P*-value[2]**
Prevotellaceae	18.5	22.3	21.7	0.65
Unidentified family 1[A]	15.3	13.1	14.8	0.65
Ruminococcaceae	9.2	10.0	9.5	0.84
Unidentified family 2[B]	8.8	9.1	7.9	0.65
Lachnospiraceae	6.0	6.3	5.9	0.84
Paraprevotellaceae	5.0	5.0	5.9	0.81
RF16	4.1	3.0	3.3	0.65
Erysipelotrichaceae	3.3	2.4	2.5	0.84
F16	2.8	4.1	2.5	0.63
BS11	2.6	2.4	3.2	0.84
Anaeroplasmataceae	2.5	1.7	2.0	0.81
Spirochaetaceae	2.0	1.5	1.6	0.74
RFP12	1.8	1.2	1.0	0.74
Veillonellaceae	1.7	2.3	2.1	0.65
S24-7	1.1	1.0	0.8	0.81
Unidentified family 3[C]	0.9	1.1	1.3	0.84
Unidentified family 4[D]	0.9	1.2	1.3	0.84
Mogibacteriaceae	0.8	0.9	1.0	0.84
Methanobacteriaceae	0.8	0.8	0.9	0.84
Unidentified family 5[E]	0.6	0.6	0.6	0.97
Clostridiaceae	0.6	0.7	0.7	0.84
Fibrobacteraceae	0.6	0.5	0.4	0.72
Victivallaceae	0.5	0.5	0.7	0.84
Other families	5.1	4.7	5.0	≥ 0.42
Unassigned OTUs	4.4	3.8	3.6	0.42

[1] CON = conventional cow-calf system without supplementation of calves. PCON = calves were creep fed. ENZ = calves were creep fed with an enhanced feed containing xylanase.

[2] Test *P*-values based on Bonferroni's method for multiple comparisons.

[A]Order *Bacteroidales*; [B]Order *Clostridiales*; [C]Phylum SR1; [D]Order RF39; [E]Order YS2.

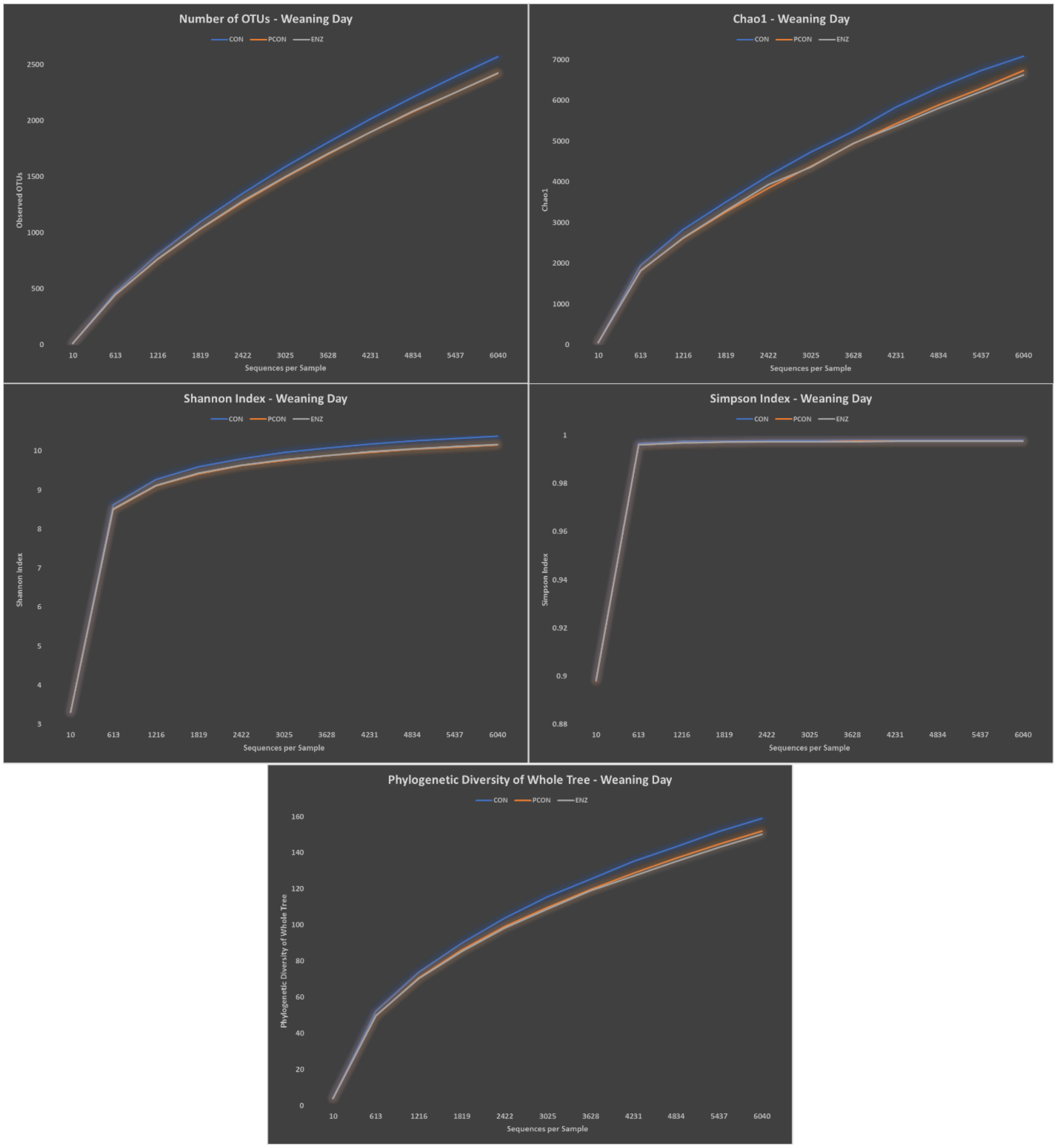

Figure S1. Alpha rarefaction plots generated using samples collected on weaning day. There is a distinction between the blue line (CON) and the other 2 lines for the majority of the traits measured, whereas the other 2 lines (orange and white – PCON and ENZ) tended to be more similar to each other, corroborating with the information presented in Table 1. *P*-values for number of OTUs, Chao1, Shannon index, Simpson index, and Phylogenetic diversity of whole tree (i.e. Faith's Phylogenetic Diversity) were 0.02, 0.05, 0.04, 0.12, and 0.01, respectively.

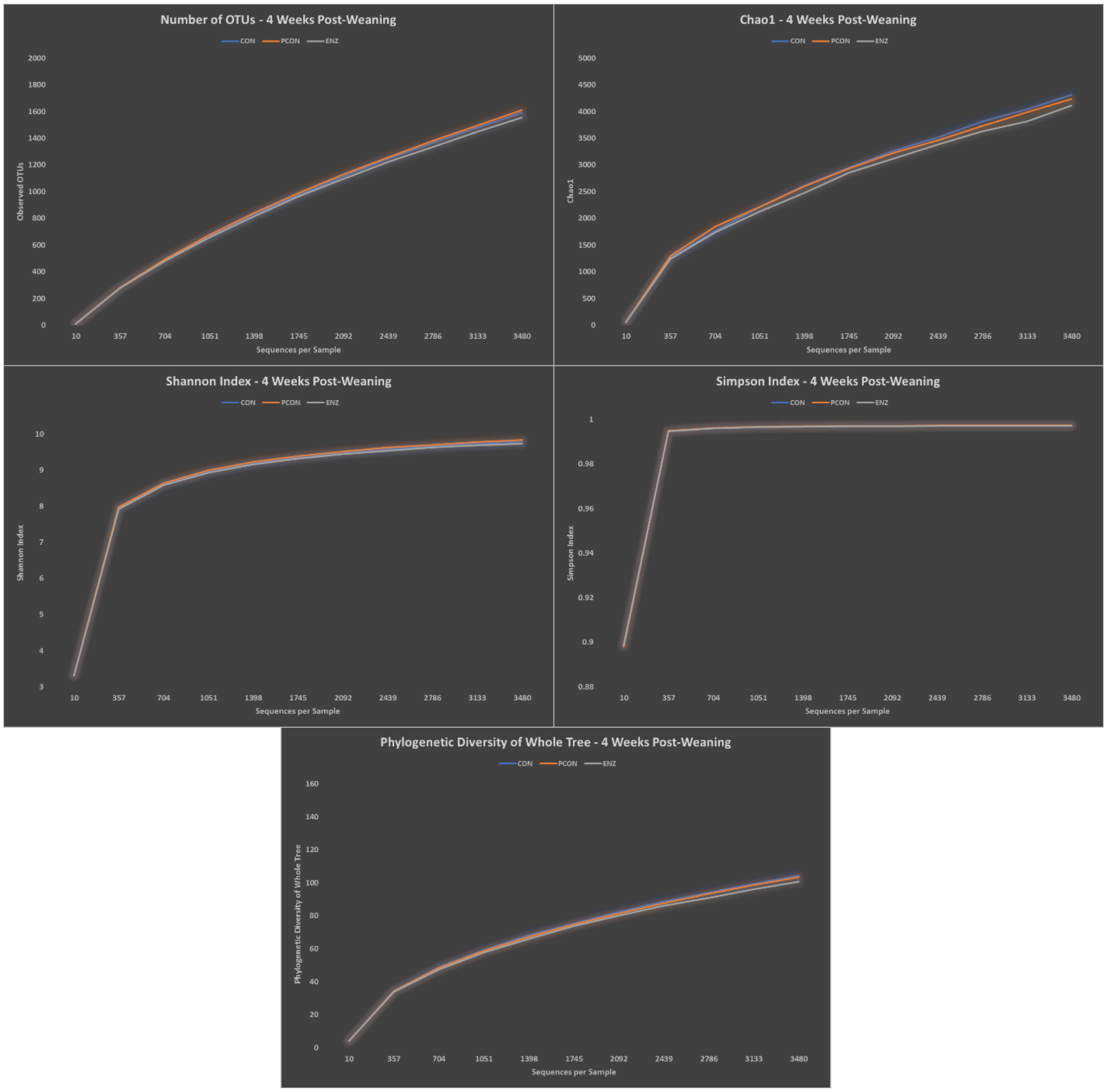

Figure S2. Alpha rarefaction plots generated using samples collected 4 weeks after calves were weaned. There are no distinction between the 3 lines (blue, orange and white – CON, PCON and ENZ) for the majority of the traits measured, corroborating with the information presented in Table 2. *P*-values for number of OTUs, Chao1, Shannon index, Simpson index, and Phylogenetic diversity of whole tree (i.e. Faith's Phylogenetic Diversity) were 0.50, 0.45, 0.58, 0.81, and 0.23, respectively.

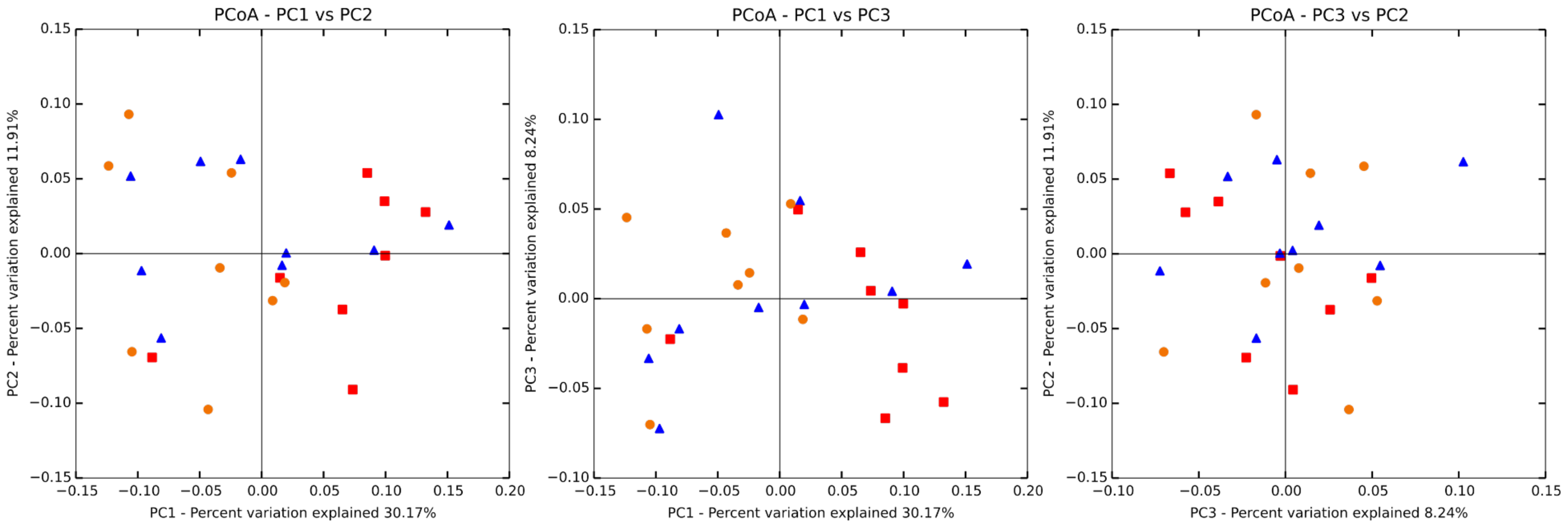

Figure S3. Principal coordinate analysis of beta diversity for samples collected on calves' weaning day using the weighted UniFrac distance matrix. ■ = Conventional cow-calf system without supplementation of calves (CON). ▲ = Group in which calves were creep fed (PCON). ● = Group in which calves were creep fed with an enhanced feed containing xylanase (ENZ). No differences due to diet were observed ($P \geq 0.99$).

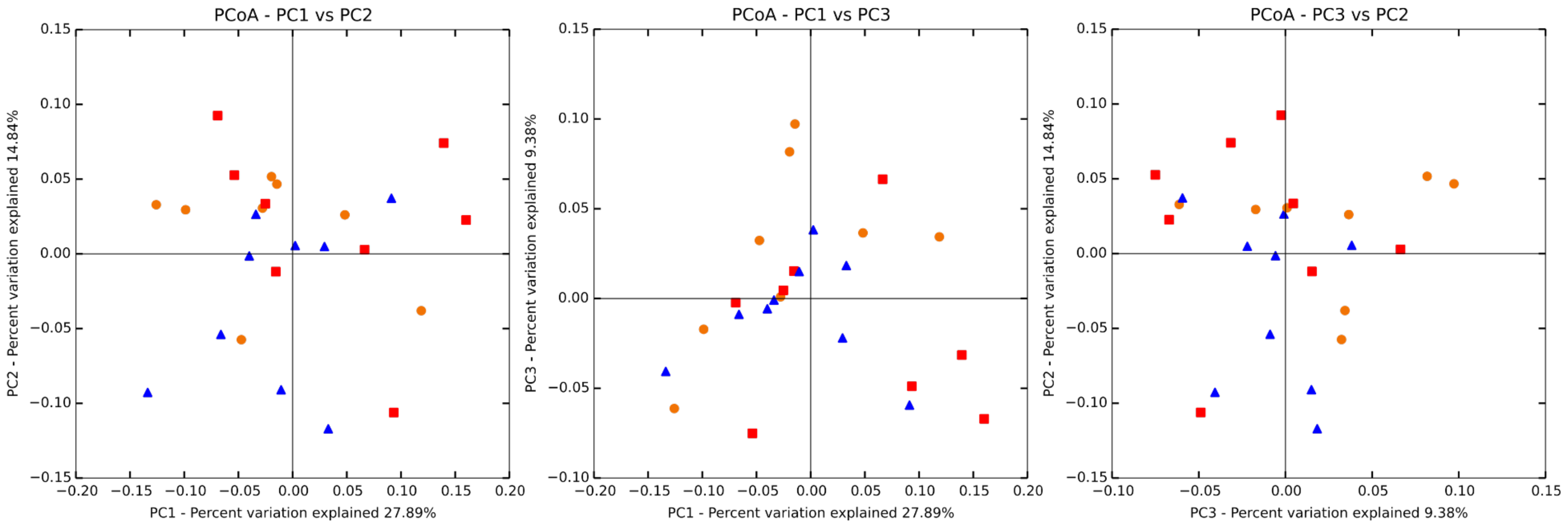

Figure S4. Principal coordinate analysis of beta diversity for samples collected 4 weeks after calves were weaned using the weighted UniFrac distance matrix. ■ = Conventional cow-calf system without supplementation of calves (CON). ▲ = Group in which calves were creep fed (PCON). ● = Group in which calves were creep fed with an enhanced feed containing xylanase (ENZ). No differences due to diet were observed ($P \geq 0.11$).

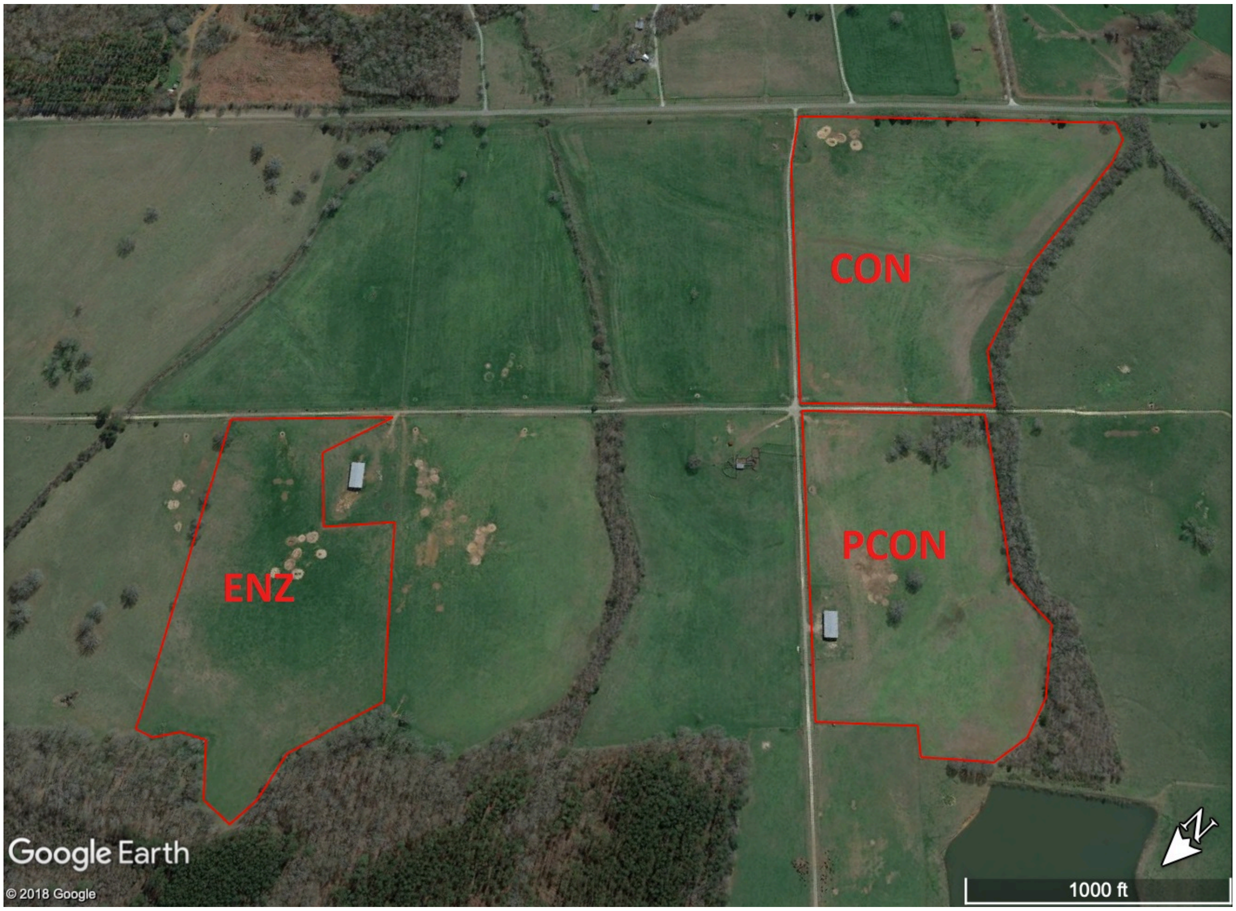

Figure S5. Aerial map showing the 3 distinct paddocks in which the cow-calf groups were located during the 105-day feeding trial. CON = conventional cow-calf system without supplementation of calves. PCON = calves were creep fed. ENZ = calves were creep fed with an enhanced feed containing xylanase.

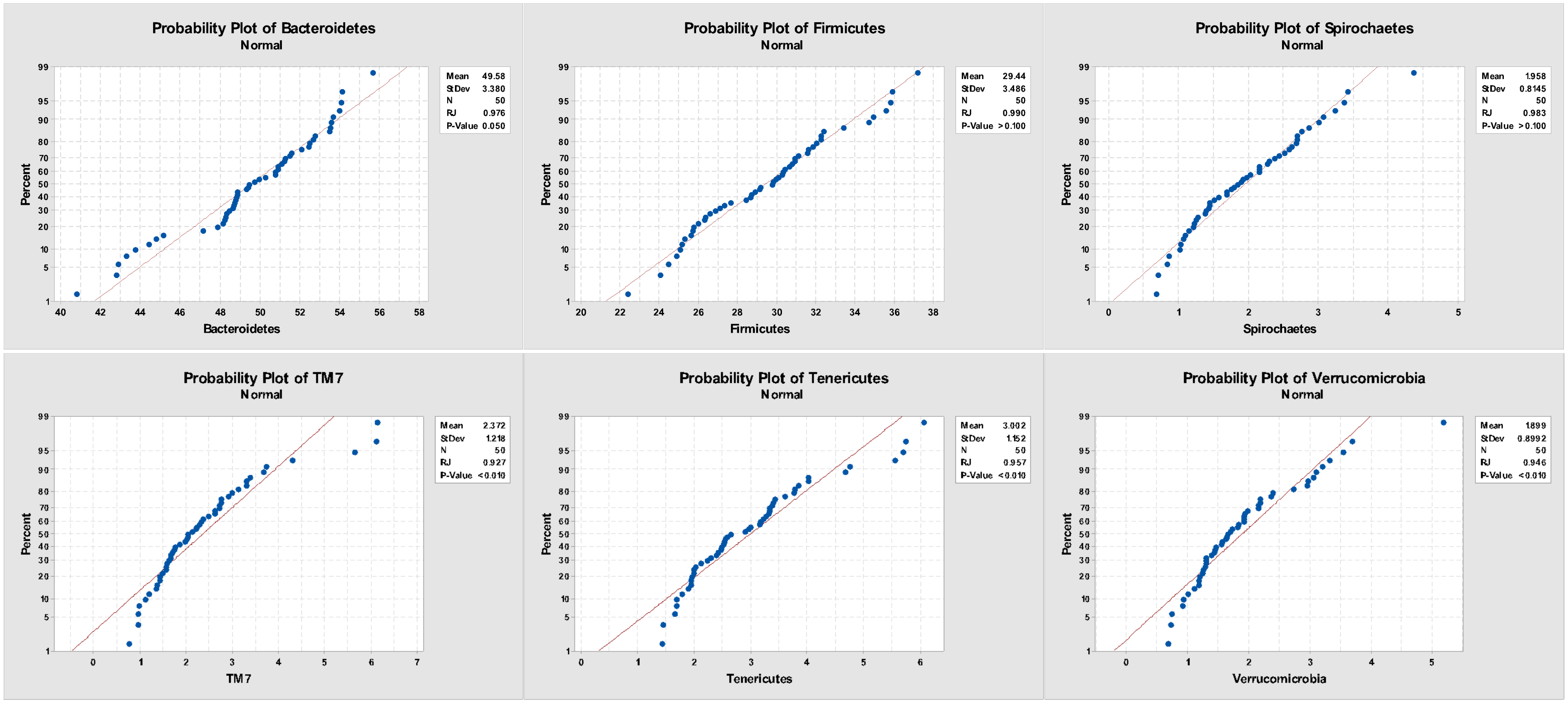

Figure S6. Normality tests* for the relative abundances of the phyla *Bacteroidetes*, *Firmicutes*, *Spirochaetes*, TM7, *Tenericutes* and *Verrucomicrobia*.

*Ryan-Joiner normality test. Probability plots shows no significant deviations from the straight line, indicating that the normal distribution is a good fit to the data.

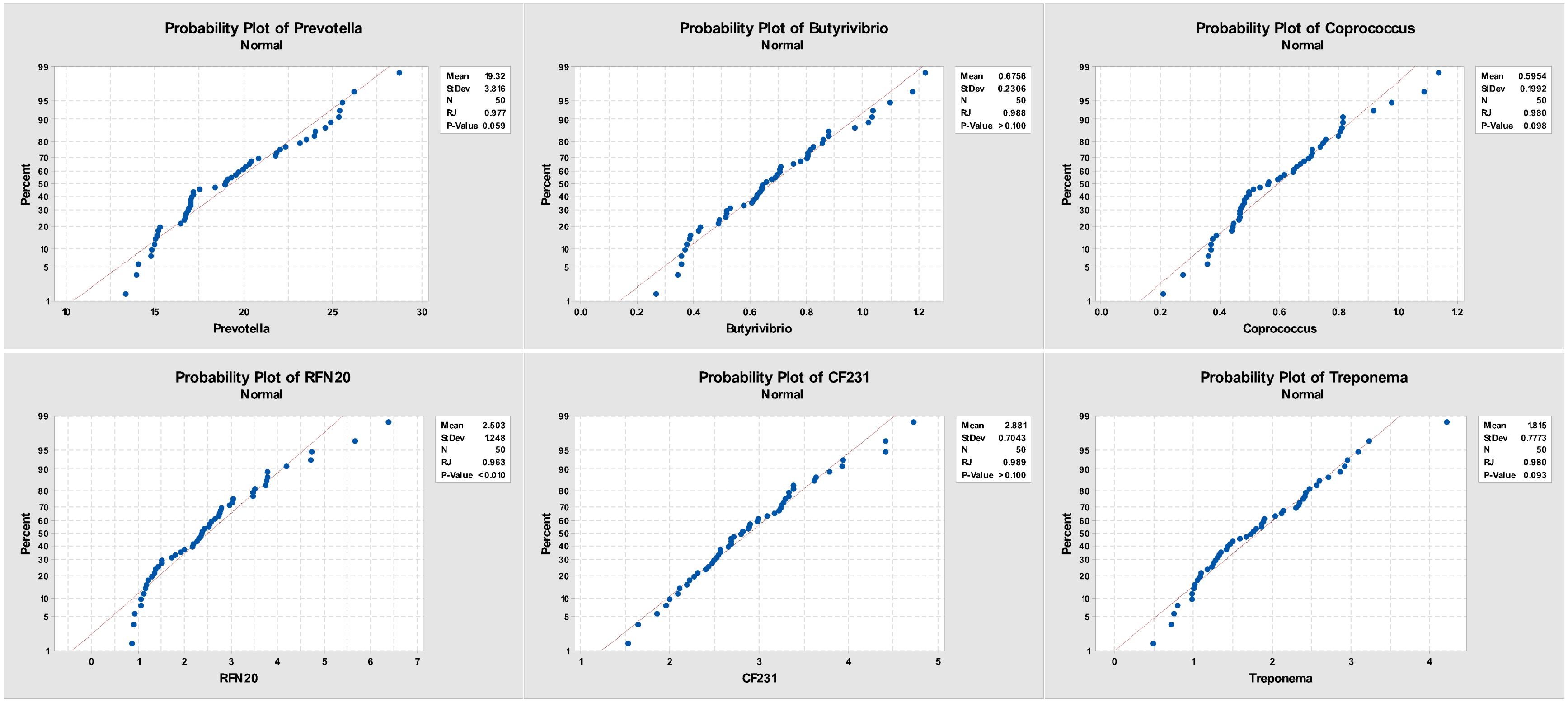

Figure S7. Normality tests* for the relative abundances of the genera *Prevotella*, *Butyrivibrio*, *Coprococcus*, RFN20, CF231, and *Treponema*.

*Ryan-Joiner normality test. Probability plots shows no significant deviations from the straight line, indicating that the normal distribution is a good fit to the data.

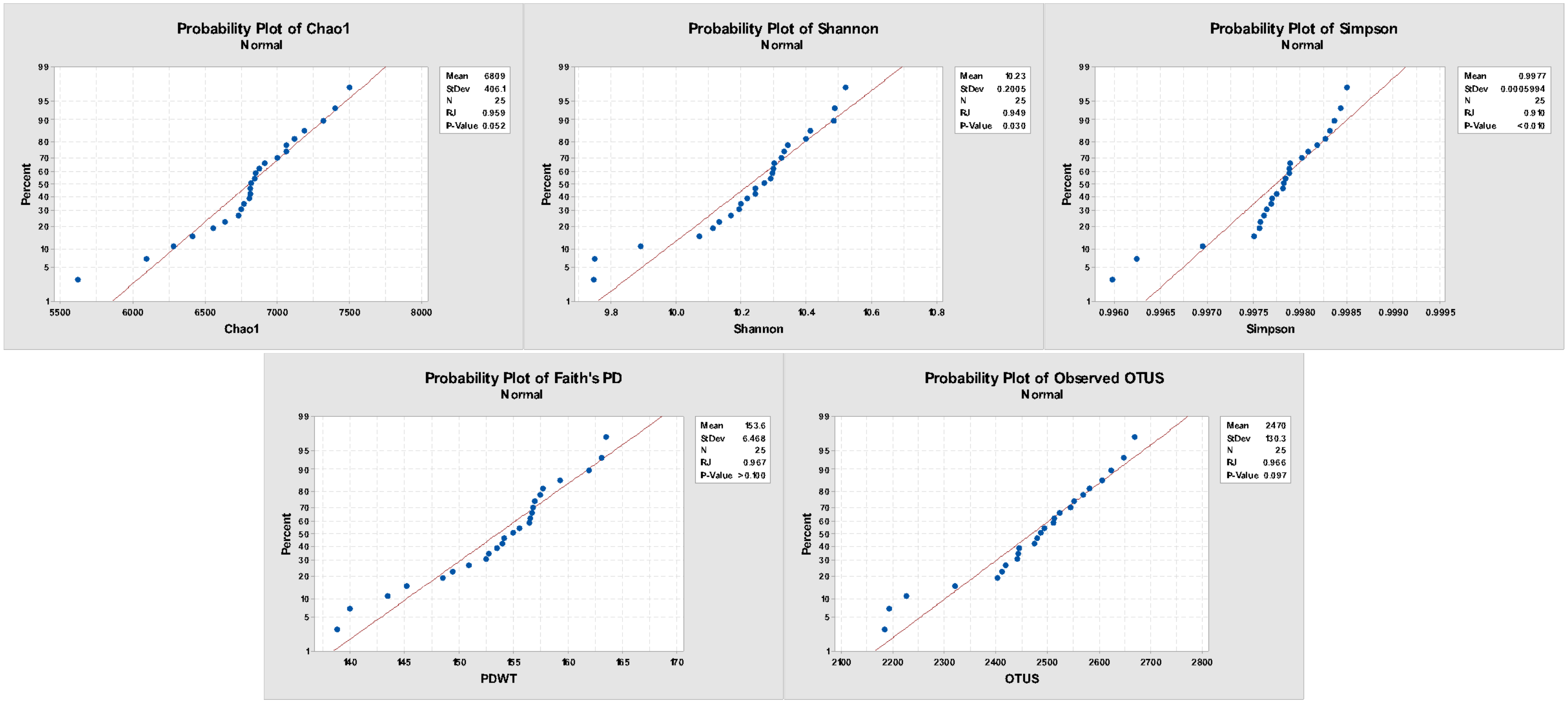

Figure S8. Normality tests* for the alpha-diversity indexes Chao1, Shannon diversity index, Simpson's diversity index, Faith's Phylogenetic Diversity, and number of observed OTUs, for samples collected on weaning day.

*Ryan-Joiner normality test. Probability plots shows no significant deviations from the straight line, indicating that the normal distribution is a good fit to the data.

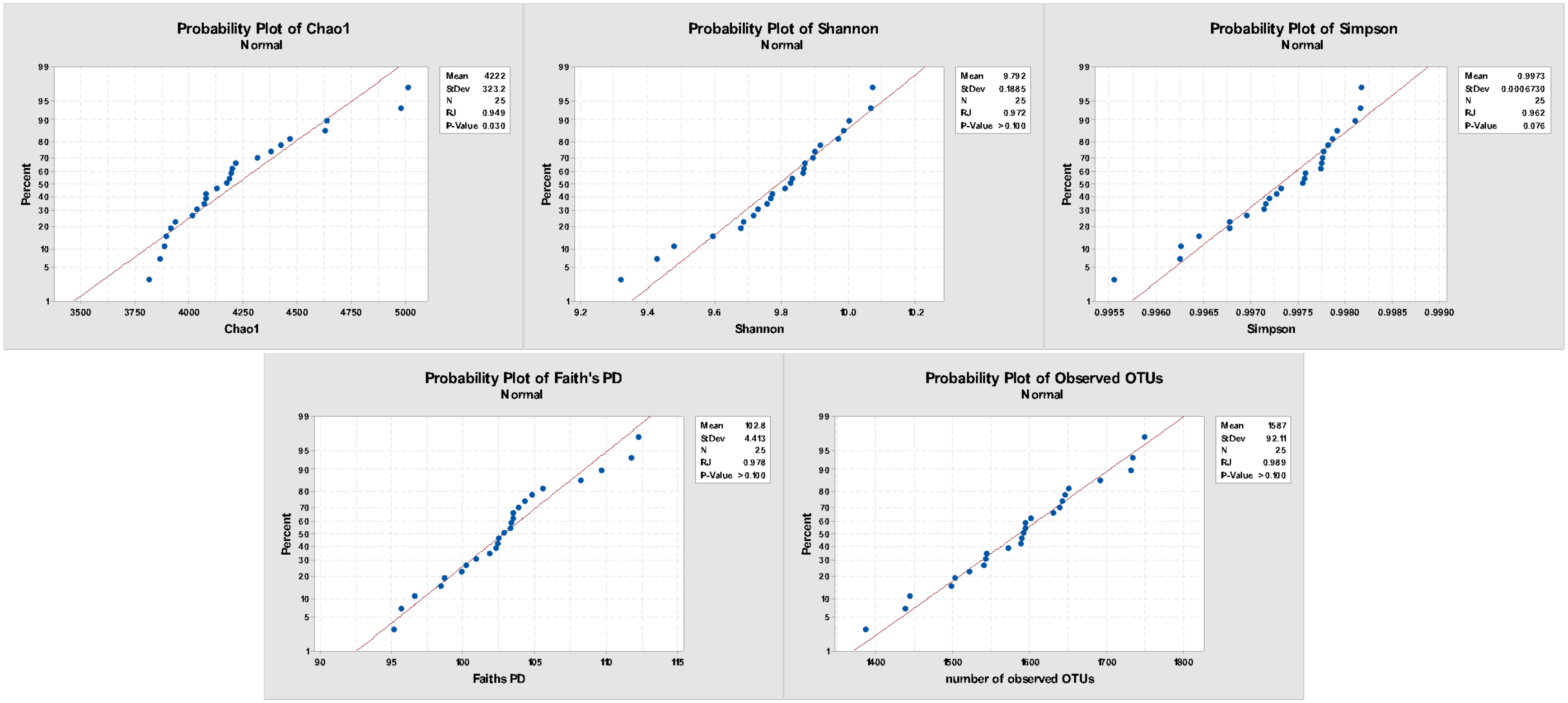

Figure S9. Normality tests* for the alpha-diversity indexes Chao1, Shannon diversity index, Simpson's diversity index, Faith's Phylogenetic Diversity, and number of observed OTUs, for samples collected 4 weeks after weaning.

*Ryan-Joiner normality test. Probability plots shows no significant deviations from the straight line, indicating that the normal distribution is a good fit to the data.

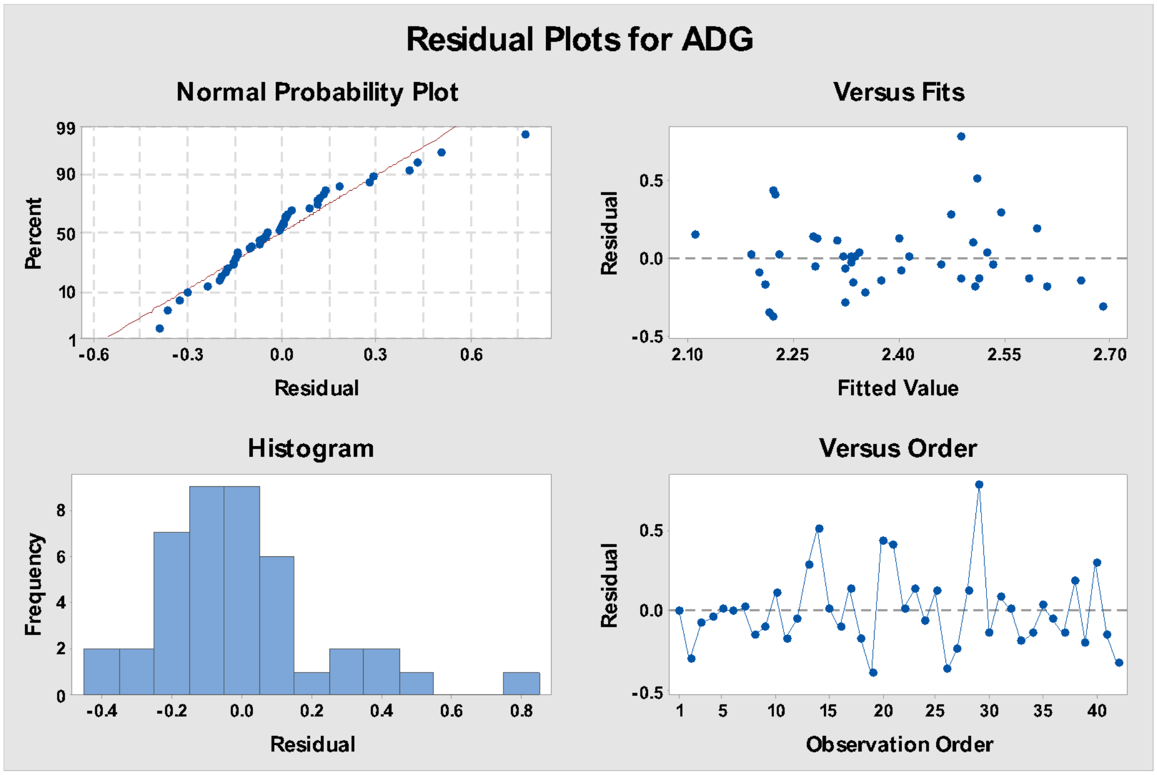

Figure S10. Residual plots to examine ANOVA goodness-of-fit for Average Daily Gain (ADG). Bell-shaped histogram and Normal Probability Plot indicate that this trait was normally distributed.
